# Extracellular Vesicle-Mediated Macrophage Polarization in Sepsis-Induced Acute Lung Injury: Molecular Mechanisms and Therapeutic Opportunities

**DOI:** 10.3390/cells15171574

**Published:** 2026-08-29

**Authors:** Yiqian Shen, Yi Tai, Xinzhe Liu, Yang Li, Zihao Zhao, Xuejun Jin, Juan Ma

**Affiliations:** 1 Key Laboratory of Natural Medicines of the Changbai Mountain, Ministry of Education, College of Pharmacy, Yanbian University, Yanji 133002, China; shenyiqian0816@163.com (Y.S.); taiyybut@163.com (Y.T.); liuxzhe@163.com (X.L.); liyang416@163.com (Y.L.); zhaozihaoyubt@163.com (Z.Z.); 2 Key Laboratory of Drug Targets and Pharmacological Screening in Colleges and Universities of Jilin Province, Molecular Medicine Research Center, Yanji 133002, China

**Keywords:** sepsis-induced acute lung injury, macrophage polarization, extracellular vesicles, immunoregulation

## Abstract

Sepsis-induced acute lung injury (SI-ALI) is a severe complication of sepsis characterized by dysregulated inflammatory responses and impaired immune homeostasis. Growing evidence indicates that extracellular vesicles (EVs), particularly exosomes, are important mediators of intercellular communication. Despite the heterogeneity of infectious sources underlying sepsis, EVs can regulate macrophage polarization and functional reprogramming by transferring diverse bioactive cargo. Consequently, EVs are involved in the pathophysiological progression of SI-ALI arising from sepsis of different etiologies. However, the mechanisms through which distinct EV cargos regulate macrophage function and contribute to SI-ALI pathogenesis remain incompletely understood. To address these issues, this review summarizes how different EV subtypes and their cargos, including RNAs, proteins, lipids, and DNA, modulate macrophage functional states through multiple signaling pathways. The effect of such processes further contributes to inflammatory reaction, immune balance, and tissue regeneration in acute lung injury caused by damage to the SI-ALI. Particularly, the EV-mediated modulation of macrophage function goes beyond the rigid M1/M2 dichotomy, being rather based on the dynamic functional repertoire involving both pro-inflammatory response and immune regulation as well as tissue regeneration. The article finally concludes with EV-based treatment approaches aimed at cargo delivery or blocking and the main problems related to translational medicine. Overall, the review article identifies the macrophage regulatory network controlled by EVs, thus helping to understand immunopathogenesis of SI-ALI as well as laying the theoretical foundation for developing EV-based precision medicine.

## 1. Introduction

Sepsis is an illness-inducing syndrome caused by a dysfunctional host reaction to infections that is characterized by immune system dysfunction, excessive inflammation, and multiple organ dysfunction. Despite many advances in the area of intensive care, sepsis is one of the main reasons for mortality in critically ill patients worldwide. Among the organs affected by sepsis, the lungs are especially vulnerable because of their specific structure and high amount of immune cells. Sepsis-induced ALI and the more severe condition called ARDS play a significant role in causing respiratory failure and death in the case of sepsis patients. ARDS may be triggered not only by sepsis but also by trauma, large transfusions [1,2]. However, in the case of sepsis, the development of ARDS is highly associated with uncontrolled infection and inflammation. From the perspective of pathophysiology, sepsis-induced ALI involves impairment of alveolar–capillary barrier function, severe inflammation, high vascular permeability, and diffuse alveolar damage. At present, treatment options for SI-ALI include measures for infection prevention, organ support, and lung protection; however, there are no treatments to address the underlying processes of immune dysfunction and tissue damage directly [3]. Therefore, elucidating the molecular mechanisms driving sepsis-induced ALI is essential for identifying novel therapeutic targets and facilitating the development of precision therapies.

Sepsis may be acquired as a result of infections at different anatomical sites and caused by different pathogens. Main sources of infections are the lungs, the abdomen, the urinary system, the bloodstream, and skin and soft tissue infections [4]. Different infection sources have their own biological characteristics and may induce different levels of inflammation, vascular dysfunction, coagulation disturbances, and immunometabolism changes [5]. However, regardless of the origin of infection or pathogen, sepsis is characterized by the dysregulation of the host response to the infection and is capable of inducing lung damage due to the development of systemic inflammation and immune dysfunction. In this process, macrophages serve as important immune cells involved in pathogen elimination, inflammation regulation, and tissue regeneration [6]. Functional activities are continuously modulated by pathogen-associated molecular patterns (PAMPs), damage-associated molecular patterns (DAMPs), cytokines, and metabolic alterations [7]. Increasing data suggest that EVs, especially exosomes, play the role of signaling vehicles for regulation of macrophage polarization and functional programming. As a result, even though different sources of infection will lead to different response patterns in the host, macrophage polarization and functional programming induced by EVs are two common and significant immune regulation mechanisms in SI-ALI of various etiologies.

However, a detailed analysis of the role of EVs in the modulation of macrophage functioning during sepsis-induced ALI is rather uncommon. Indeed, previous studies focused mainly on regulatory effects exerted by the cargo of individual EVs; however, the impact of various subpopulations of EVs and their diverse cargos on the functions of macrophages was not extensively studied [6,7]. Modulation of macrophage activity by EVs takes place in several pathological processes, such as inflammation, tissue injury, barrier dysfunction, and cell death. In addition, the cargo of EVs may affect the functioning of macrophages to varying degrees depending on the septic microenvironment. It is still unclear how much of the relative importance of molecules in exosomes in regulating the course of disease is determined by the local microenvironment of the immune response. At the same time, most current studies still make use of the classical M1/M2 classification that does not allow for a full representation of the heterogeneity and plasticity of macrophages in SI-ALI. Such an approach is crucial in developing a better understanding of macrophage regulation via EVs. Although there has been considerable interest in using EVs as therapeutic agents and carriers, problems with in vivo distribution, targeting, and production still stand in the way of clinical application.

To address these knowledge gaps, this review focuses on EV-mediated macrophage polarization and functional reprogramming in SI-ALI. We first discuss the functional heterogeneity and dynamic changes in macrophages during SI-ALI. We then summarize the molecular mechanisms through which distinct EV subtypes and their functional cargoes regulate macrophage states and examine the relative contributions of different cargoes under diverse septic microenvironmental conditions. On this basis, we analyze how EV-mediated macrophage regulation affects inflammatory and immune homeostasis, lung tissue repair, alveolar–capillary barrier integrity, and programmed cell death. Finally, we discuss potential therapeutic strategies involving the targeting of pathogenic EVs and the application of protective and engineered EVs.

## 2. Macrophage Polarization in Sepsis-Induced ALI

Macrophages are vital effector cells of the innate immune system and exert central functions in pathogen elimination, inflammatory modulation and tissue homeostasis maintenance. Resident macrophages in lung tissue mainly consist of AMs and interstitial macrophages. AMs primarily reside in the alveolar space and form the first immune barrier of the lung against exogenous pathogens; they are responsible for recognizing, phagocytosing and clearing inhaled pathogens while regulating local inflammatory responses [8]. Interstitial macrophages are primarily distributed in the pulmonary stroma and perivascular regions, and their main functions are to maintain immune homeostasis, regulate inflammation, and mediate tissue remodeling [9]. Despite their differences in anatomical distribution and functional orientation, both populations exhibit prominent functional plasticity and jointly participate in the initiation and progression of sepsis-induced ALI via synergistic regulation of immune responses [10].

Dysregulated macrophage function constitutes a key component of immune disorder in SI-ALI. The canonical M1/M2 model generally categorizes macrophages into pro-inflammatory M1 and anti-inflammatory M2 phenotypes, providing a simplified framework for describing macrophage responses under diverse stimulatory conditions. Nevertheless, this dichotomy fails to fully capture the complexity of macrophages in vivo. M1 and M2 mainly represent prototypical activation states established under defined experimental conditions. Within tissue microenvironments, by contrast, macrophages are concurrently exposed to multiple stimuli including pathogen-associated signals, cytokines, damage-associated signals, and metabolic alterations, thereby adopting cellular states with distinct transcriptional and functional signatures. Beyond canonical M1-like and M2-like phenotypes, mixed and intermediate states that simultaneously exhibit pro-inflammatory, immunoregulatory, or tissue-repair features can also exist [11,12]. M0 primarily serves as a reference state in vitro without specific polarizing stimulation and does not correspond to a stably existing independent macrophage subset in vivo. Accordingly, strictly classifying macrophages into two mutually exclusive M1 or M2 types is insufficient to reflect their functional states in vivo. Specifically, the functional state of macrophages represents a dynamic functional program co-regulated by multiple signaling pathways, which can be continuously remodeled along with disease progression and shifts in the local microenvironment.

This heterogeneity is even more pronounced in the disease progression of SI-ALI. Following infection, PAMPs and DAMPs can rapidly activate pulmonary macrophages. Pro-inflammatory signaling pathways—such as TLR/NF-κB, MAPK, and NLRP3—are amplified, leading to the subsequent release of inflammatory mediators such as TNF-α, IL-1β, and IL-6 [13]. At this stage, pro-inflammatory functions are typically predominant, and cells may exhibit classic M1-like characteristics. However, M1-like macrophages are not the only type present in the early stages of infection. Pro-inflammatory and anti-inflammatory responses in sepsis can be triggered simultaneously at an early stage [14,15], and macrophages with M2 or immunoregulatory characteristics may also be present concurrently. Thus, there are no strict temporal boundaries between different functional programs, and their relative intensities may vary depending on the infection burden, the extent of tissue damage, and the local immune environment.

As the pro-inflammatory response continues to intensify, inflammatory mediators released by macrophages can promote the recruitment and activation of neutrophils. This exacerbates damage to the alveolar epithelium and vascular endothelium, leading to disruption of the alveolar–capillary barrier, pulmonary edema, and impaired gas exchange [16]. Furthermore, in a CLP-induced sepsis model, an enhanced pro-inflammatory phenotype of alveolar macrophages is associated with increased pulmonary inflammation and tissue damage [16]. Additionally, combined stimulation with LPS and IFN-γ can similarly induce pronounced M1-like characteristics and sustain the expression of pro-inflammatory mediators [17]. Therefore, while the pro-inflammatory function of macrophages contributes to pathogen clearance and host defense, their persistent or excessive activation can also amplify pulmonary inflammation and exacerbate tissue damage.

**Figure 1:**
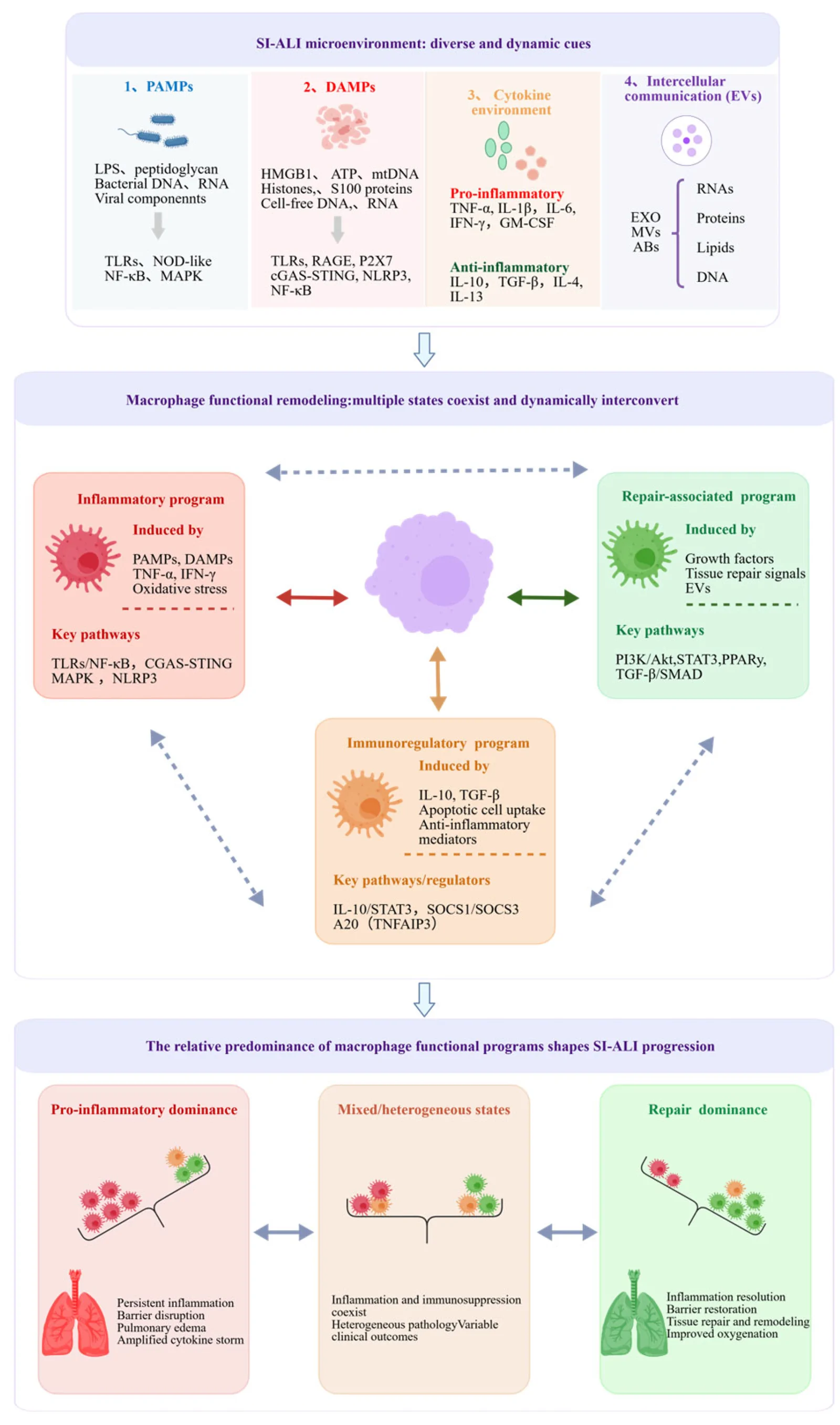
Dynamic changes in macrophage polarization during the progression of sepsis-associated ALI. Blue downward arrows show the sequence of pathological events, while double-headed arrows indicate reversible transitions between functional states. The red and green lung icons represent lung injury and recovery, respectively.

As sepsis progresses, the macrophage microenvironment undergoes continuous changes, resulting in dynamic adaptations in macrophage phenotype and function. The persistent presence of PAMPs and DAMPs sustains pro-inflammatory signaling, while IL-10, TGF-β, and signals for the clearance of apoptotic cells help enhance immunoregulatory and tissue repair processes. During this phase, some macrophages retain strong pro-inflammatory characteristics, while others increase the production of immunoregulatory factors such as IL-10 and Arg1. When the latter are relatively enhanced, macrophages can help limit excessive inflammation, clear apoptotic cells, and promote lung tissue repair [18]. Furthermore, if the infection is brought under control and tissue damage gradually subsides, these functions will facilitate the resolution of inflammation and the restoration of the alveolar–capillary barrier. If the infection persists or the immune imbalance is not corrected, persistent inflammation and ongoing tissue damage may occur. Thus, the macrophage responses that are observed in SI-ALI cannot be sufficiently characterized by a discrete or definite transition threshold between M1 and M2 polarization. Instead, macrophages have dynamically controlled functional programs of pro-inflammatory responses, immune regulation, and tissue repair during disease progression, as shown in Figure 1.

Based on this, the following discussion of EV-mediated macrophage regulation in this review is not based solely on the traditional M1/M2 polarization paradigm. Rather, it takes into account the overall functional impact of EVs on macrophages, such as the control of inflammatory reactions, immune homeostasis, and tissue repair. This functional view can offer a more detailed insight into the multifaceted and dynamic role of EV-macrophage interactions in SI-ALI.

## 3. Biology and Immunoregulatory Properties of EVs

EVs are a heterogeneous population of membrane-bound vesicles that vary in their biogenesis, molecular composition, and biological properties. The cell of origin and the microenvironmental conditions affect their composition and functional properties. By exchanging bioactive molecules, various EV populations can regulate the activity of recipient cells and thus affect a broad spectrum of physiological and pathological processes. Therefore, this section first provides an overview of the major types of EVs and their biological characteristics, followed by a focus on the biological properties and immunomodulatory functions of exosomes, laying the foundation for further exploration of the mechanisms by which EVs mediate the regulation of macrophage polarization.

### 3.1. Classification of EVs

Based on their formation mechanisms, sources, and biological characteristics, EVs are generally classified into several major types, including exosomes, microvesicles (MVs), and apoptotic bodies [19].

Exosomes are one of these populations, due to their well-defined biogenesis, comparatively well-characterized molecular composition, and wide functional applicability. Exosomes are therefore the most researched type of EV in studies examining EV-mediated processes in SI-ALI. Exosomes are small EVs that are mainly formed by the endosomal pathway through the formation of multivesicular bodies (MVBs). In exosome biogenesis, the plasma membrane invaginates to form early endosomes, which then develop into late endosomes and MVBs. The following merging of MVBs with the plasma membrane discharges intraluminal vesicles into the extracellular space, which are known as exosomes. Exosomes typically measure between 30 and 150 nm in diameter and contain a lipid bilayer membrane that is highly enriched with various functional biomolecules, such as proteins, lipids, and nucleic acids. These properties allow exosomes to be involved in intercellular communication and to regulate the biological activities of recipient cells [20].

Microvesicles are primarily formed through direct budding from the cell membrane; they are typically larger than exosomes, with a diameter of approximately 100–1000 nm. Unlike exosomes, the formation of microvesicles relies more heavily on cell membrane remodeling and changes in the cytoskeleton. Related studies have shown that microvesicles can carry membrane proteins, lipids, and nucleic acid molecules. They are also involved in processes such as the regulation of inflammatory responses, cell migration, and changes in vascular function [21,22,23].

Apoptotic bodies primarily originate from membrane-bound structures formed during the process of programmed cell death. They have a diameter of approximately 1–5 µm and may contain fragments of organelles, DNA, proteins, and other components. Although research on apoptotic bodies in SI-ALI is currently limited, their potential role in the clearance of dead cells, the establishment of immune tolerance, and the regulation of inflammation is gradually attracting attention [24].

During sepsis-associated acute lung injury (ALI), infectious stimuli and changes in the inflammatory microenvironment can induce immune cells, alveolar epithelial cells, and vascular endothelial cells to release EVs. These EVs can carry bioactive molecules such as nucleic acids, proteins, and lipids, and influence the recruitment of inflammatory cells, the regulation of immune responses, and the process of lung tissue repair [25,26,27,28].

Various types of EVs are involved in intercellular communication and can regulate the function of recipient cells by carrying functional cargo such as proteins, lipids, and nucleic acids. However, current research on EV-mediated immune regulation in sepsis-associated acute lung injury remains primarily focused on exosomes. Because exosomes originate from the endosomal-multivesicular body pathway, their biogenesis and related regulatory mechanisms have been studied in considerable depth; they also possess a relatively stable membrane structure and a well-established identification system. Numerous studies have shown that exosomes can participate in the regulation of immune cell function by delivering various functional molecules. In particular, they play a crucial role in the inflammatory response and phenotypic remodeling of macrophages [25,29,30,31]. Therefore, the remainder of this paper will focus on EV-mediated regulation of macrophage polarization, with an emphasis on analyzing the mechanisms by which the cargo of exosomes influences macrophage functional remodeling.

### 3.2. Biological Characteristics of Exosomes

Exosomes are currently the most extensively studied subtype of EVs. Their biogenesis, membrane structure, and cargo composition collectively determine their intercellular delivery and regulatory characteristics [32]. Exosomes possess a lipid bilayer membrane that protects their molecular cargos from enzymatic degradation, thereby enhancing their stability and enabling long-distance intercellular communication. Exosomes encapsulate a variety of bioactive cargo, including miRNAs, lncRNAs, circRNAs, proteins, and lipids [33,34,35]. The composition of these cargoes varies depending on the source and physiological state of the donor cells, and this variation confers different biological characteristics on the exosomes.

Although CD9, CD63, CD81, TSG101, and Alix remain the most widely used markers for exosome identification [36]. However, recent research has shifted from phenotypic characterization to the functional heterogeneity of cargos within exosomes. Recent research indicates that the biological effects of exosomes are not only related to their surface characteristics but are also influenced by the composition of their internal functional cargo and the patterns of its interactions.

### 3.3. Immunoregulatory Functions of Exosomes

Exosomes serve not only as vectors for intercellular communication but also as key regulators of the immune microenvironment. By delivering a variety of bioactive cargo, they coordinate innate and adaptive immune responses. They are also involved in various pathological processes, including inflammation regulation, immune homeostasis, and tissue repair [37,38]. Unlike soluble cytokines, exosomes can simultaneously transport multiple functional molecules, enabling synergistic regulation of target cells. This results in more sustained and comprehensive immunomodulatory effects [39,40].

The molecular cargo which is being carried by the exosomes dynamically shifts during the course of inflammatory diseases. This allows the donor cells to carefully control nearby and distant target cells. Immune cell-derived exosomes have been known to modulate inflammatory cell recruitment and cytokine production. Endothelial cell-derived exosomes participate in endothelial barrier function, while mesenchymal stem cell-derived exosomes have mainly anti-inflammatory, anti-apoptotic, and tissue-reparative effects [41,42]. All these different populations of exosomes create a very intricate communication network between cells. This network not only regulates the immune system but also repairs tissue and maintains physiological homeostasis.

Among the multiple target cells influenced by exosomes, macrophages have emerged as central mediators of exosome-induced immune remodeling. Owing to their remarkable phenotypic plasticity, macrophages rapidly respond to exosome-derived molecular signals by dynamically altering their polarization status. Consequently, exosome-mediated macrophage polarization profoundly influences inflammatory cytokine production, immune cell recruitment, tissue regeneration, and programmed cell death, establishing macrophages as a critical regulatory hub linking intercellular communication to immune remodeling during sepsis-induced ALI.

Recent studies have also shown that the biological activities of exosomes are not just governed by a single cargo but by the cooperative effects of multiple bioactive molecules. The various molecules in exosomes, including RNA, proteins, DNA, and lipids, contribute to the precise tuning of M1 and M2 polarization. They work together on important signaling pathways such as SIRT1/SOCS1, NF-κB, PI3K/Akt, STAT3, and the NLRP3 inflammasome pathway [43]. Together, these signaling networks are coordinately regulated by various components of exosomes. They regulate the initiation and resolution of inflammation, repair of the alveolar–capillary barrier, and lung tissue regeneration. All of these factors eventually contribute to the course of ALI due to sepsis [44,45].

**Figure 2:**
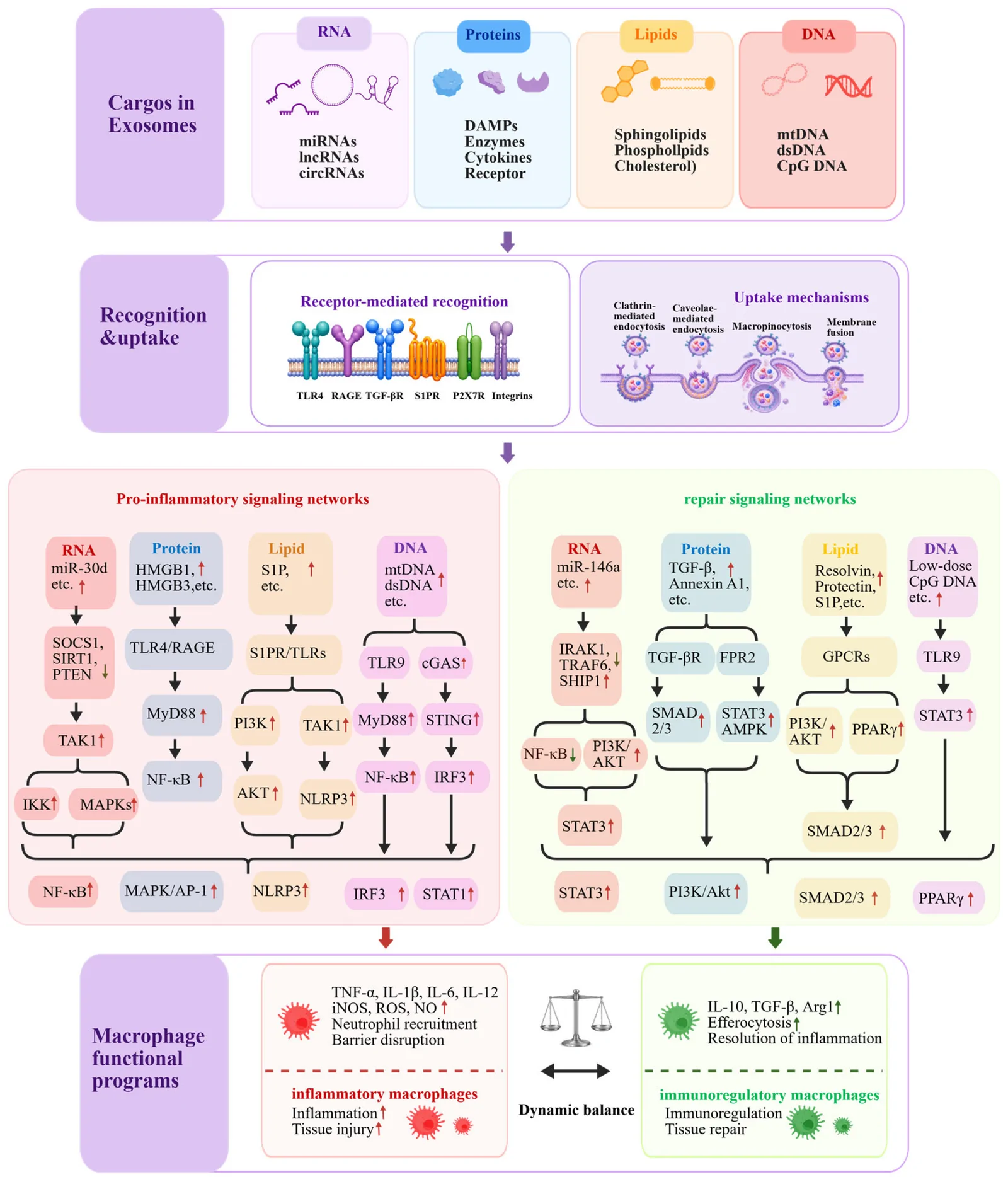
Exosomes involved in regulating macrophage polarization: source and cargos. In the figure, red and green denote pro-inflammatory and repair-related signaling, respectively. Downward arrows trace the pathway from exosome uptake to macrophage responses. The ↑ and ↓ symbols indicate increases and decreases in expression or activity. The scale reflects the balance between inflammatory and immunoregulatory macrophage programs.

Taken together, the immunoregulatory functions of exosomes arise from the coordinated actions of multiple bioactive cargos rather than individual molecules, as shown in Figure 2. Therefore, elucidating how distinct cargos in exosomes collectively orchestrate macrophage polarization is essential for understanding the immunopathogenesis of sepsis-induced ALI and for advancing exosome-based precision therapies.

The following sections therefore systematically summarize the molecular mechanisms by which RNAs, proteins, and lipids in exosomes regulate M1/M2 macrophage polarization. Particular emphasis is placed on how these distinct cargos coordinately modulate inflammatory homeostasis, lung tissue repair, alveolar–capillary barrier integrity, and programmed cell death, thereby establishing an integrated mechanistic framework linking exosome-mediated macrophage polarization to the progression of sepsis-induced ALI and its therapeutic implications.

## 4. Mechanisms by Which EVs Regulate Macrophage Polarization

In sepsis-associated acute lung injury, EVs participate in macrophage functional remodeling by delivering various functional components. They also regulate inflammatory responses and tissue repair processes. Therefore, this section first analyzes the molecular mechanisms by which different components of exosomes regulate macrophage polarization. Next, we discuss the roles of microvesicles and apoptosomes in macrophage immunoregulation, as well as EV-mediated macrophage functional remodeling, to identify potential therapeutic targets for sepsis-associated ALI.

### 4.1. Mechanisms of Macrophage Polarization Regulated by Various Cargoes in Exosomes

#### 4.1.1. RNA in Exosomes Regulates Macrophage Polarization

MiRNAs are among the most extensively investigated functional cargos of exosomes and play pivotal roles in intercellular communication by post-transcriptionally regulating gene expression in recipient cells. In the pulmonary microenvironment, exosomal miRNAs involved in regulating macrophage function can be derived from a variety of cells, including pulmonary epithelial cells, immune and immunoregulatory cells, as well as mesenchymal stem/stromal cells. During sepsis-induced ALI, exosomes released from different cell types deliver specific miRNAs to macrophages, thereby regulating macrophage polarization through inflammatory signaling pathways and reshaping the pulmonary immune microenvironment [39,46,47,48].

Several pro-inflammatory miRNAs in exosomes have been identified as potent inducers of M1 macrophage polarization. Among these, miR-30d-5p from neutrophil-derived exosomes is one of the most thoroughly studied examples. After being taken up by macrophages, miR-30d-5p directly inhibits *SOCS1* and *SIRT1*, leading to sustained activation of the NF-κB signaling pathway. In addition to promoting M1 polarization, miR-30d-5p induces macrophage pyroptosis, thereby enhancing the release of pro-inflammatory cytokines such as IL-1β and IL-18. These events amplify the inflammatory cascade, exacerbate alveolar–capillary barrier disruption, and ultimately aggravate ALI [49,50].

Besides miR-30d-5p, other miRNAs in exosomes also contribute to pro-inflammatory macrophage polarization. For instance, exosomal miR-92a-3p facilitates M1 polarization via the NF-κB signaling pathway. It is linked to enhanced inflammatory mediator production and aggravated lung inflammation [51]. Moreover, additional pro-inflammatory miRNAs delivered by immune cell-derived exosomes have been shown to sustain NF-κB activation and reinforce inflammatory responses, thereby accelerating disease progression [52].

Collectively, although these pro-inflammatory miRNAs target distinct downstream molecules, they ultimately converge on NF-κB-centered inflammatory signaling networks, promoting M1 macrophage polarization, inflammatory amplification, and lung injury. These results indicate that interfering with the pathogenic miRNAs in exosomes or their downstream signaling might be a promising therapeutic approach. This may inhibit excessive inflammation and reduce ALI caused by sepsis.

Besides the pro-inflammatory miRNAs, there is growing evidence that various other exosomal miRNAs induce M2 polarization of macrophages. This helps to prevent excessive inflammatory responses and promote tissue remodeling in ALI associated with sepsis [53]. One of the most extensively studied therapeutic exosome populations is that derived from mesenchymal stem cells (MSCs).It induces M2 macrophage polarization and decreases the generation of pro-inflammatory cytokines, such as TNF-α and IL-1β. It also increases the secretion of anti-inflammatory mediators, such as IL-10. All these effects act cumulatively to decrease pulmonary inflammation and enhance lung function [54,55]. MSC-derived exosomes also deliver miR-150-5p, which promotes M2 polarization through modulation of targeting *Irs1*, thereby suppressing inflammatory responses and alleviating alveolar injury. Likewise, miR-184-3p facilitates M2 macrophage polarization by regulating Sema7a-mediated signaling, contributing to the restoration of alveolar–capillary barrier integrity and accelerating lung tissue repair [56,57,58,59].

Beyond MSC-derived exosomes, exosomes released from other cell types also exhibit anti-inflammatory properties. miRNAs from exosomes derived from immune cells or tissue-resident cells promote M2 polarization while inhibiting inflammation associated with M1 polarization through signaling pathways such as STAT3 and PI3K/Akt, thereby facilitating the resolution of inflammation and tissue regeneration [60,61]. Importantly, emerging evidence suggests that M2 macrophages not only suppress excessive inflammation but also promote fibroblast regulation, alveolar epithelial regeneration, restoration of alveolar–capillary barrier integrity, and reconstruction of the local immune microenvironment. Accordingly, therapeutic strategies aimed at enhancing M2 macrophage polarization have attracted increasing attention for the treatment of sepsis-induced ALI [62].

Collectively, although individual miRNAs in exosomes target distinct downstream molecules, they converge on signaling pathways including PI3K/Akt, IRS1, and STAT3 to promote M2 macrophage polarization and coordinately mediate anti-inflammatory responses, tissue repair, and restoration of alveolar–capillary barrier function. These regulatory miRNAs, along with pro-inflammatory miRNAs leading to M1 polarization, form a dynamic exosome-mediated macrophage polarization network. This network is involved in immune remodeling during the course of sepsis-induced ALI. Overall, these results provide additional support for the development of exosome-based miRNA therapeutics to achieve precision lung injury intervention in the realm of inflammation.

Exosomes also contain various types of non-coding RNAs, such as lncRNAs and circRNAs, in addition to miRNAs. Although none of these RNAs are translated into proteins, they have tremendous regulatory influence on gene expression. This occurs through transcriptional regulation, competing endogenous RNA (ceRNA) networks, and intracellular signaling pathways. Consequently, they have emerged as important regulators of inflammatory responses, immune homeostasis, and tissue repair [63].

In recent years, lncRNAs in exosomes have gained increasing attention as critical modulators of macrophage polarization. Unlike miRNAs, which primarily suppress target gene expression directly, lncRNAs frequently regulate macrophage phenotypes by functioning as ceRNAs or by modulating inflammatory signaling pathways. Once lncRNAs in exosomes are delivered to recipient macrophages, they regulate signaling pathways such as NF-κB, STAT3, and PI3K/Akt, thereby influencing the balance between M1 and M2 polarization and promoting the progression of sepsis-induced ALI [64,65]. Among them, *TUG1* competitively sequesters pro-inflammatory miRNAs to modulate downstream inflammatory signaling pathways, thereby driving macrophage polarization toward the M2 phenotype. It reduces the expression of pro-inflammatory factors such as TNF-α and IL-1β while elevating the secretion of anti-inflammatory mediators including IL-10, ultimately alleviating pulmonary inflammation and facilitating tissue repair [66,67].

Macrophage polarization has also been implicated in other lncRNAs, such as *Gm16023* (or the lncRNA identified in the original study) and *lncRNA-Cox2.* These molecules control inflammatory signaling pathways and immune-related gene expression, thus inhibiting M1 polarization or enhancing M2 polarization to mitigate inflammatory damage in sepsis-induced ALI [68,69,70,71].

Overall, distinct classes of non-coding RNAs in exosomes function cooperatively rather than independently to establish a multilayered regulatory network governing macrophage polarization. These coordinated RNA networks collectively regulate inflammatory homeostasis, repair of the alveolar–capillary barrier, and tissue regeneration. Thus, non-coding RNAs in exosomes may serve as therapeutic targets for the treatment of sepsis-induced ALI.

#### 4.1.2. Proteins in Exosomes Regulating Macrophage Polarization

The exosomal RNA mainly controls the post-transcriptional expression of genes, whereas exosomal proteins are direct signaling effectors and can quickly alter the reaction of recipient cells. There is growing evidence that proteins contained in exosomes directly stimulate or inhibit inflammatory signaling pathways, thus controlling macrophage polarization, inflammatory responses, tissue repair and alveolar–capillary barrier homeostasis in sepsis-induced ALI [72,73].

Among the pro-inflammatory proteins identified to date, high mobility group box 1 (HMGB1) is one of the best-characterized mediators. HMGB1 in exosomes interacts with TLR4 and the receptor for advanced glycation end products (RAGE) to sustain NF-κB activation, thereby promoting M1 macrophage polarization and inducing robust production of pro-inflammatory cytokines, including TNF-α, IL-1β, and IL-6. These events amplify inflammatory cascades and exacerbate pulmonary epithelial and endothelial injury [74,75,76]. In addition, exosomes released from activated inflammatory cells transport other pro-inflammatory proteins that further reinforce inflammatory signaling and accelerate disease progression [77]. Conversely, increasing attention has been directed toward the protective effects of proteins delivered by mesenchymal stem cell (MSC)-derived exosomes. Among these, heat shock protein B8 (HSPB8) has emerged as a representative anti-inflammatory mediator. Delivery of HSPB8 in exosomes suppresses excessive inflammatory responses, promotes M2 macrophage polarization, and alleviates histopathological lung injury. Likewise, proteins such as CXCL14 contribute to immune remodeling by facilitating M2 polarization and enhancing tissue repair during ALI [78,79,80].

On the whole, despite the various molecular properties of different exosome proteins, they all eventually affect the inflammatory signaling pathways, thus controlling the polarization of macrophages. M1 polarization and inflammatory amplification are mainly promoted by pro-inflammatory proteins, and M2 polarization, tissue regeneration, and restoration of alveolar–capillary barrier integrity are promoted by protective proteins [81]. Thus, exosomal proteins are likely to be important as carriers in the regulation of ALI caused by sepsis.

#### 4.1.3. Lipids in Exosomes Regulating Macrophage Polarization

Besides RNAs and proteins, lipids are another vital category of bioactive cargos in exosomes. Even though the lipids in exosomes were originally considered to be mainly structural cargos that serve to preserve membrane integrity and to mediate membrane fusion, there is growing evidence that they also serve as bioactive signaling molecules that mediate intercellular communication. Lipids in exosomes have become significant regulators of macrophage polarization in sepsis-induced ALI by regulating inflammatory signaling, cellular metabolism, and immune responses [82,83].

Of the lipid species found in exosomes, sphingosine-1-phosphate (S1P), sphingosine, and ceramide have been most studied. S1P, as a strong bioactive lipid mediator, triggers downstream signaling pathways, such as PI3K/Akt and STAT3, by interacting with S1P receptors. Therefore, S1P facilitates M2 macrophage polarization, inhibits excessive production of inflammatory cytokines, improves alveolar epithelial repair, and helps to restore alveolar–capillary barrier integrity [84,85]. Sphingosine has several other regulatory functions besides exosome biogenesis. Persistent inflammatory activation has been linked to dysregulated sphingolipid metabolism, and recovery of sphingosine homeostasis suppresses inflammatory damage and facilitates tissue repair. Similarly, ceramide, a major structural lipid in exosome biogenesis, also plays a role in inflammatory signaling and cell fate control. The over-accumulation of ceramides has been associated with increased inflammatory reactions and tissue damage in ALI [86,87,88].

Notably, the lipids in exosomes not only serve as signaling cargo that is delivered to recipient cells, but also regulate exosome biosynthesis, cargo loading, membrane fusion, and cellular uptake. Therefore, lipid metabolism influences both the quantity and biological activity of exosomes, suggesting that lipid-targeted interventions may simultaneously modulate exosome production and immune regulatory capacity. In summary, various lipid molecules participate in macrophage phenotypic switching, alveolar–capillary barrier repair and tissue regeneration via co-regulating inflammatory signaling, immune metabolism and cell membrane homeostasis [89].

#### 4.1.4. DNA in Exosomes Regulates Macrophage Polarization

In addition to RNA, proteins, and lipids, DNA is also one of the key functional components that can be detected in exosomes. Recent studies have shown that exosomes can carry genomic DNA (gDNA) and mitochondrial DNA (mtDNA) and participate in the regulation of immune function in recipient cells by mediating the transfer of nucleic acid information [90].

In the inflammatory microenvironment of sepsis-associated ALI, infectious stimuli, oxidative stress, and mitochondrial damage can promote the release of mtDNA-rich EVs from damaged cells [91]. Upon entering macrophages, exogenous mtDNA is recognized as a damage-associated molecular pattern by TLR9 and DNA-sensing pathways such as cGAS-STING. It activates the NF-κB transcriptional program and NLRP3 inflammasome signaling, promoting the release of pro-inflammatory factors such as IL-1β, IL-6, and TNF-α, thereby enhancing the inflammatory activation of macrophages [92]. For example, oxidized mtDNA can directly promote NLRP3 inflammasome activation and induce IL-1β maturation, demonstrating that mtDNA is a key endogenous signaling molecule linking mitochondrial damage to the inflammatory response [93]. Furthermore, the release of mitochondrial DNA can activate the cGAS-STING-mediated innate immune response, promoting the expression of inflammation-related genes [94]. Therefore, in SI-ALI, mtDNA-rich exosomes may enhance macrophage innate immune recognition, promote the maintenance of a pro-inflammatory phenotype, and participate in the amplification of the inflammatory response.

Nevertheless, the biological impact of EV-DNA depends on a complex of factors, such as the origin of the DNA, the exosome composition, and the disease stage. Comparatively, DNA is mainly an immunological danger signal, which changes the inflammatory condition of macrophages by stimulating certain sensing pathways. Thus, DNA in exosomes is generally a controller that connects cellular damage signals to the immune response, but not the polarization of macrophages.

#### 4.1.5. Synergistic Regulatory Mechanisms of Macrophage Polarization by Multiple Components in Exosomes

**Figure 3:**
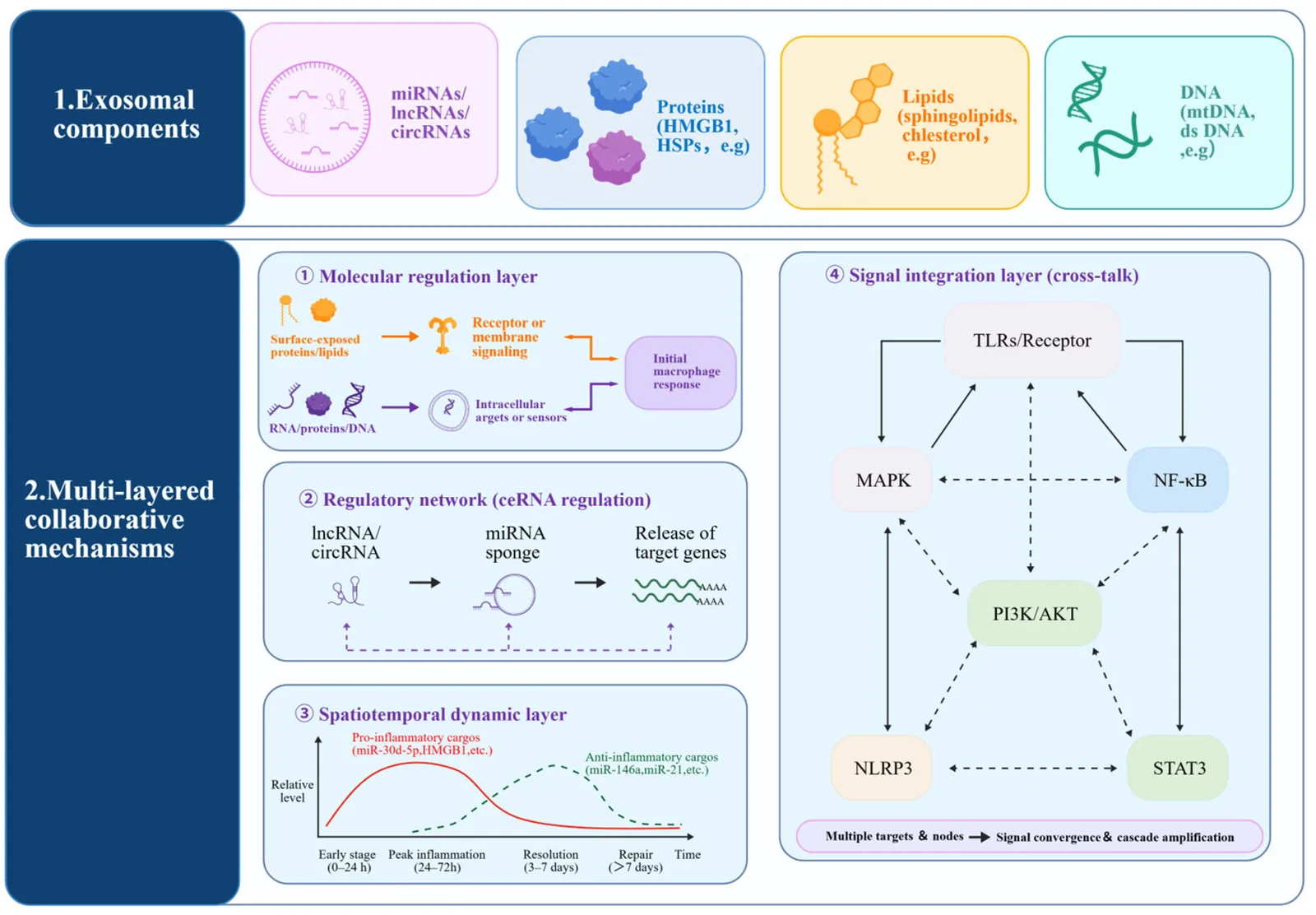
Multicomponent Synergistic Regulatory Mechanisms of Macrophage Polarization. In the figure, in the molecular regulation layer, orange arrows indicate signals generated by the interaction of vesicle surface proteins or lipids with receptors or the cell membrane, while purple arrows indicate the action of RNA, protein, or DNA cargo after it enters the cell. The purple dashed lines in the ceRNA network indicate competitive regulation between lncRNAs/circRNAs, miRNAs, and target genes. The red and green curves in the Spatiotemporal Dynamics layer represent the relative levels of pro-inflammatory and anti-inflammatory molecules at different stages, respectively. In the Signal Integration layer, the colors of each node are used solely for differentiation and do not indicate activation, inhibition, or expression levels.

Although the RNA, proteins, DNA, and lipids in exosomes can independently regulate macrophage polarization, these different bioactive components do not act in isolation. Instead, they jointly determine macrophage fate and the progression of sepsis-induced ALI through a multi-level and multi-dimensional regulatory network, as illustrated in Figure 3.

At the molecular regulation layer, distinct cargos in exosomes regulate recipient macrophages through complementary mechanisms. miRNAs primarily repress target gene expression at the post-transcriptional level, whereas lncRNAs and circRNAs modulate gene expression through ceRNA networks. Proteins in exosomes can exert their effects through ligand–receptor interactions, protein transfer, or intracellular signaling. Lipids in exosomes serve not only as bioactive signaling molecules but also help maintain the membrane structure of exosomes and influence the interaction between the vesicles and recipient cells. Unlike the components mentioned above, DNA in exosomes primarily serves as a damage-associated signal and participates in the activation of the innate immune response through DNA-sensing pathways such as TLR9 and cGAS-STING. It can thus be seen that the various components are not identical in terms of their binding sites and modes of regulation. This is also the reason why they exhibit different functional efficacies in receptor-expressing macrophages.

At the regulatory network layer, interactions among different classes of non-coding RNAs establish a multilayered regulatory network. Through ceRNA-mediated interactions, miRNAs, lncRNAs, and circRNAs coordinately regulate inflammatory gene expression, enabling dynamic and context-dependent modulation of immune responses. This type of network organization increases the strength and accuracy of exosome-mediated signaling.

The biological activities of exosome-carrying cargos at the spatiotemporal dynamic layer change during disease progression. In the case of a preponderance of inflammatory signals and tissue damage signals in the pulmonary microenvironment, pro-inflammatory RNA, inflammation-related proteins, or mtDNA may have a stronger immunostimulatory effect. Components that have anti-inflammatory or metabolic regulatory effects can have stronger functional effects when signals associated with immune regulation and tissue repair are increased. Nevertheless, this shift does not mean that SI-ALI is simply in a linear transition between a pro-inflammatory and immunosuppressive or reparative state.

Overall, exosomely modulated polarization status of macrophages cannot be ascribed to a single molecular mediator or a specific signal transduction pathway. Instead, this phenotype is determined by concerted biological actions of multifarious bioactive constituents that are packaged into exosomes, i.e., RNAs, polypeptides and lipid moieties. These cargos together regulate inflammatory responses and tissue repair through coordinated molecular regulation, signaling crosstalk, and intercellular communication. A deeper understanding of these complex regulatory networks may provide new insights into the pathogenesis of sepsis-induced ALI and facilitate the development of novel therapeutic strategies targeting exosome-mediated immune regulation.

#### 4.1.6. Priority of Each Cargo Within Exosomes in Different Sepsis Microenvironments

Existing studies have shown that under live-bacterial infection conditions, exosomes derived from infected macrophages can induce inflammatory responses in uninfected macrophages. Proteinase K treatment significantly attenuates this effect, whereas RNase or DNase treatment exerts no comparable influence. This indicates that proteins serve as the major pro-inflammatory effector components under such conditions [95]. During the progression of sepsis, stimulation by LPS and CLP increases the levels of eCIRP in macrophage-derived exosomes. Compared with exosomes containing eCIRP, exosomes lacking eCIRP show a significantly reduced ability to induce TNF-α and IL-6 production in macrophages. This suggests that this protein is an important functional component mediating the pro-inflammatory effects of exosomes [96].

In contrast, component-depletion experiments revealed that the roles of proteins and RNA in EVs depend on the state of the recipient macrophages. In LPS-unstimulated macrophages, proteins of exosomes primarily contribute to inflammatory activation. In LPS-stimulated macrophages, however, RNA primarily helps limit excessive inflammatory responses [97]. The experiment by Zheng et al. also validated this view. They found that in LPS-stimulated macrophages, exosomes derived from MSCs and pretreated with LPS exerted their protective effects primarily through RNA. This protective effect was significantly reduced following treatment with RNase, whereas treatment with Proteinase K had a minimal impact [61]. Overall, under conditions of pathogen infection, proteins in exosomes play a crucial role in inducing inflammatory activation in macrophages. However, when the recipient macrophage is already in an LPS-induced inflammatory state, specific RNAs will limit or regulate excessive inflammatory responses. These observations indicate that the relative priority of RNA- and protein-mediated regulatory functions depends on the state of the donor cell and its recipient macrophage.

Thus, the sepsis microenvironment plays a crucial role in defining the relative functional roles of the cargo in exosomes. This effect can be exerted in two main forms: first, by changing the relative concentrations of various cargoes in donor cells and, second, by changing the response state of recipient macrophages.

To begin with, the microenvironment of sepsis may affect the loading of exosome components by changing the condition of donor cells. These Cargos in exosomes are not mere copies of the contents of the donor cell; they vary dynamically in composition and concentration in response to the environment of the cell. As an illustration, hypoxia may affect exosome biogenesis and loading of different components via HIF, Rab proteins and oxidative stress-mediated processes, and may alter the miRNA expression profile in EVs. Of these, miR-23a, miR-21, and miR-223 all show environmental variation [98]. Plasma exosomes from patients with septic shock had a different RNA profile from those of healthy controls. Several miRNAs remained altered after 7 days of treatment, and myeloperoxidase (MPO) mRNA was still elevated. These findings suggest that in-flammation and oxidative stress during sepsis may have sustained effects on exosomal RNA cargo [99]. In addition, inflammatory stimulation can also reshape the protein composition of exosomes. Studies have shown that following LPS stimulation, 341 proteins increased and 363 proteins decreased in macrophage-derived exosomes [100]. Furthermore, cellular stress can also affect mitochondrial-related components in exosomes; Antimycin A-induced mitochondrial oxidative stress significantly increases the mtDNA content in exosomes [101]. By comparing macrophage-derived exosomes under different stimulation conditions, studies have found significant differences in the composition of proteins and small non-coding RNAs [102]. These results indicate that even when derived from the same cell type, exosomes produced under different inflammatory and metabolic conditions will differ in their levels of RNA, proteins, and other components. These variations will affect the potential of RNA, proteins, lipids, and DNA to participate in the regulation of recipient cells.

However, the concentration of exosome components does not directly reflect their functional contributions. Currently, most studies conduct bulk analysis of large numbers of EVs; the results obtained reflect the average composition of the EV population and do not adequately capture the specific components carried by individual EVs [103]. Current research has shown that a given miRNA is not present in all exosomes, and there are significant differences in the types and quantities of RNA carried by different exosomes. Therefore, it is difficult to effectively determine which cargo plays the primary role based solely on the concentrations of the various components in the exosomes.

On the other hand, whether the components within exosomes can produce strong functional effects also depends on the response state of the receptor macrophages. PAMPs, DAMPs, and cytokines in sepsis can remodel macrophage receptor expression, metabolic pathways, and intracellular signaling activity. Therefore, even when mediated by exosomes with similar cargo, macrophages in different states may elicit different responses.

The RNA in exosomes typically must undergo uptake by recipient cells, intracellular transport, and cargo release before reaching the appropriate subcellular locations to exert its regulatory effects [104]. Its effects depend not only on the amount effectively delivered but are also influenced by the abundance of the target RNA and the state of the relevant signaling networks. When specific RNA is effectively delivered to target cells, the functional contribution of the RNA is enhanced. This explains why the effects of the RNA components were significantly enhanced following LPS stimulation in the aforementioned study.

The function of protein components is influenced by their topological location and receptor expression status [105]. Some proteins located on the surface of exosomes can bind directly to macrophage membrane receptors [106]. They can initiate downstream signaling without requiring the release of intracellular components. Therefore, compared to components that rely on endocytosis, cytoplasmic release, and target recognition, these surface proteins may offer the advantage of more direct and rapid signal initiation. However, faster signal initiation does not necessarily mean that these proteins always play a dominant role in the final functional effects. This is due to the fact that ongoing macrophage functional remodeling may still be co-regulated by RNA and other cargo.

Similar receptor-dependent mechanisms may also exist in DNA and lipid cargo. The effects of exosomal DNA depend on the activity of DNA-sensing pathways such as TLR9 and cGAS–STING. Under conditions of heightened oxidative stress or mitochondrial dysfunction, if receptor-expressing macrophages also possess high DNA-sensing capacity, the effects of immunostimulatory DNA may be further amplified. The effects of lipid cargo, on the other hand, are closely related to lipid receptors and metabolic pathways. For example, the cytokine environment can alter the expression of lipid metabolism-related molecules in macrophages, such as PPARγ and CD36 [107], thereby influencing their response to lipid signals. Therefore, the functional contribution of the cargo depends not only on what the exosomes carry but also on whether the recipient macrophages are in a state capable of responding to those components.

**Figure 4:**
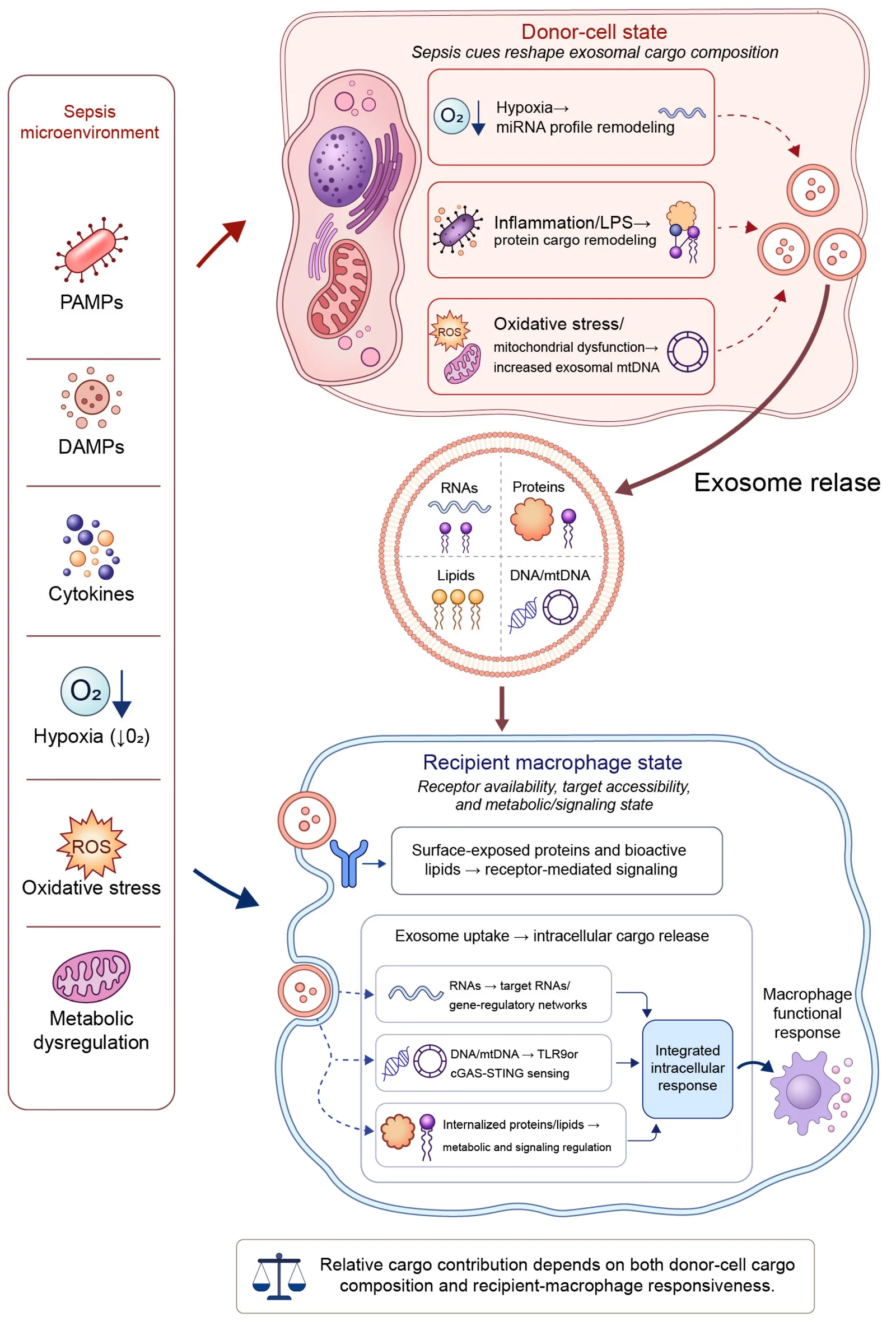
Sepsis microenvironment shapes the relative functional contribution of cargos in Exosomes. In the figure, red arrows trace changes in donor cells, exosomal cargo, and exosome release under septic conditions. Blue arrows show the effects on recipient macrophages. Solid arrows mark the direction of each process, whereas dashed arrows indicate cargo loading and intracellular delivery.

In summary, the relative contributions of exosome components—such as RNA, proteins, lipids, and DNA—to immunomodulatory effects are not fixed but are distinctly dependent on the microenvironment. The sepsis microenvironment can alter the loading, relative abundance, and molecular modifications of these components. It can also reshape the metabolic state and signaling responsiveness of receptor macrophages, thereby influencing the actual regulatory roles of the various components, as illustrated in Figure 4.

### 4.2. Regulation of Macrophage Polarization by Microvesicles

In addition to exosomes, MVs—another important subtype of EVs—also participate in the functional remodeling of immune cells during the SI-ALI process. Because MVs are directly derived from the process of cell membrane budding, their compositional characteristics reflect the activation status of the donor cells, thereby enabling them to rapidly respond and transmit signals within the inflammatory microenvironment. During SI-ALI, various injury-associated cells and immune cells can release MVs, which influence the inflammatory response and functional state transition of macrophages by delivering a variety of bioactive molecules.

During sepsis-associated ALI, infectious stimuli, the release of inflammatory cytokines, and oxidative stress can significantly promote the release of MVs by immune cells, platelets, and pulmonary vascular endothelial cells. MVs generated under inflammatory conditions can carry various molecules associated with immune activation and participate in the regulation of the inflammatory state of macrophages. Previous studies have shown that MVs derived from alveolar macrophages are involved in the progression of ALI inflammation. Furthermore, LPS stimulation can induce alveolar macrophages to release large quantities of MVs. These MVs can carry inflammation-related molecules and promote inflammatory responses in lung tissue, thereby exacerbating alveolar injury [108]. Furthermore, once inflammation-derived MVs enter recipient macrophages, they can activate innate immune signaling pathways such as TLR4/NF-κB, thereby enhancing inflammatory transcriptional programs. They also promote the release of inflammatory cytokines such as TNF-α, IL-1β, and IL-6, thereby sustaining the inflammatory activation of macrophages.

However, the regulation of macrophage function mediated by MVs is not limited to pro-inflammatory effects but is highly dependent on the type of donor cell and the disease microenvironment. MVs derived from immunoregulatory cells or cells involved in tissue repair can reduce the inflammatory response of macrophages by modulating signaling pathways such as NF-κB, PI3K/Akt, and STAT3, and promote the expression of repair-related molecules such as IL-10 and Arg1 [109].

The effects of MVs on macrophage functional states exhibit distinct source-dependent and phase-specific characteristics. In the early stages of SI-ALI, inflammatory MVs released by injury-associated cells may contribute to the amplification of inflammatory signals and immune dysregulation. In contrast, during the resolution phase of inflammation, MVs derived from repair-associated cells promote the recovery of macrophage function and the restoration of lung tissue homeostasis. Therefore, MVs do not unidirectionally drive macrophages toward a specific phenotype, but rather participate in the dynamic remodeling of macrophages by regulating inflammatory signaling and cellular functional states at different pathological stages.

### 4.3. Apoptotic Bodies Regulate the Functional Reprogramming of Macrophages

During SI-ALI, persistent infectious stimuli and inflammatory damage can induce programmed cell death in alveolar epithelial cells, vascular endothelial cells, and immune cells, and promote the formation and release of ABs. Unlike exosomes and microvesicles, ABs primarily originate from dying cells. Their biological functions are more closely associated with signal transduction from dying cells, immune recognition, and clearance processes [110]. Therefore, ABs-mediated intercellular communication plays a unique role in regulating the inflammatory response of macrophages and the restoration of tissue homeostasis.

The recognition and phagocytosis of apoptotic cells (ABs) by macrophages are key mechanisms for maintaining immune homeostasis in the lungs. Apoptosis-related recognition signals on the surface of ABs, such as phosphatidylserine (PS), can be recognized by phagocytic receptors on the surface of macrophages. These include members of the TAM receptor family (Tyro3, Axl, and MerTK) as well as receptors of the TIM family [111,112]. This process not only promotes the clearance of dead cells but also induces macrophages to produce anti-inflammatory and tissue-repair-related factors such as IL-10 and TGF-β. At the same time, it suppresses the release of pro-inflammatory mediators such as TNF-α and IL-1β, thereby limiting persistent inflammatory responses and promoting the repair of tissue damage [113,114,115]. Studies have shown that phagocytic processes mediated by receptors such as MerTK can induce metabolic and transcriptional remodeling in macrophages. Furthermore, these processes involve immune-regulatory pathways such as PI3K/Akt and PPARγ, thereby promoting the expression of genes associated with anti-inflammation and tissue repair [116,117].

In addition to promoting the clearance of dead cells, ABs can also influence the functional state of receptor macrophages by delivering molecules associated with their source cells. Studies have shown that ABs can carry components such as cell membrane lipids, nucleic acids, and intracellular proteins. Furthermore, after being taken up by macrophages, ABs can participate in the processes of immune tolerance and resolution of inflammation [118]. In particular, PS derived from apoptotic cells not only serves as a key signal for macrophages to recognize apoptotic structures but also promotes the initiation of anti-inflammatory programs by activating phagocytic receptor-related pathways such as MerTK and Axl. Furthermore, nucleic acids and lipid metabolism-related molecules released by apoptotic cells can further influence the transcriptional regulation and metabolic state of macrophages, thereby contributing to the termination of inflammatory responses and tissue repair processes [119]. In addition, nucleic acids released by apoptotic cells, as well as molecules associated with lipid metabolism, can further influence the transcriptional regulation and metabolic state of macrophages. Furthermore, these factors can participate in the resolution of inflammatory responses and tissue repair processes [120,121]. Therefore, ABs are not merely products of cell death. Instead, they are a class of functional vesicular structures that reflect the state of tissue damage and participate in immune regulation.

In summary, ABs primarily participate in SI-ALI immune remodeling by regulating macrophage phagocytic capacity, the inflammatory resolution process, and tissue repair responses. This provides a new avenue for EV-mediated regulation of macrophage function.

## 5. The Impact of EV-Mediated Macrophage Functional Reprogramming on the Pathological Progression of SI-ALI

**Figure 5:**
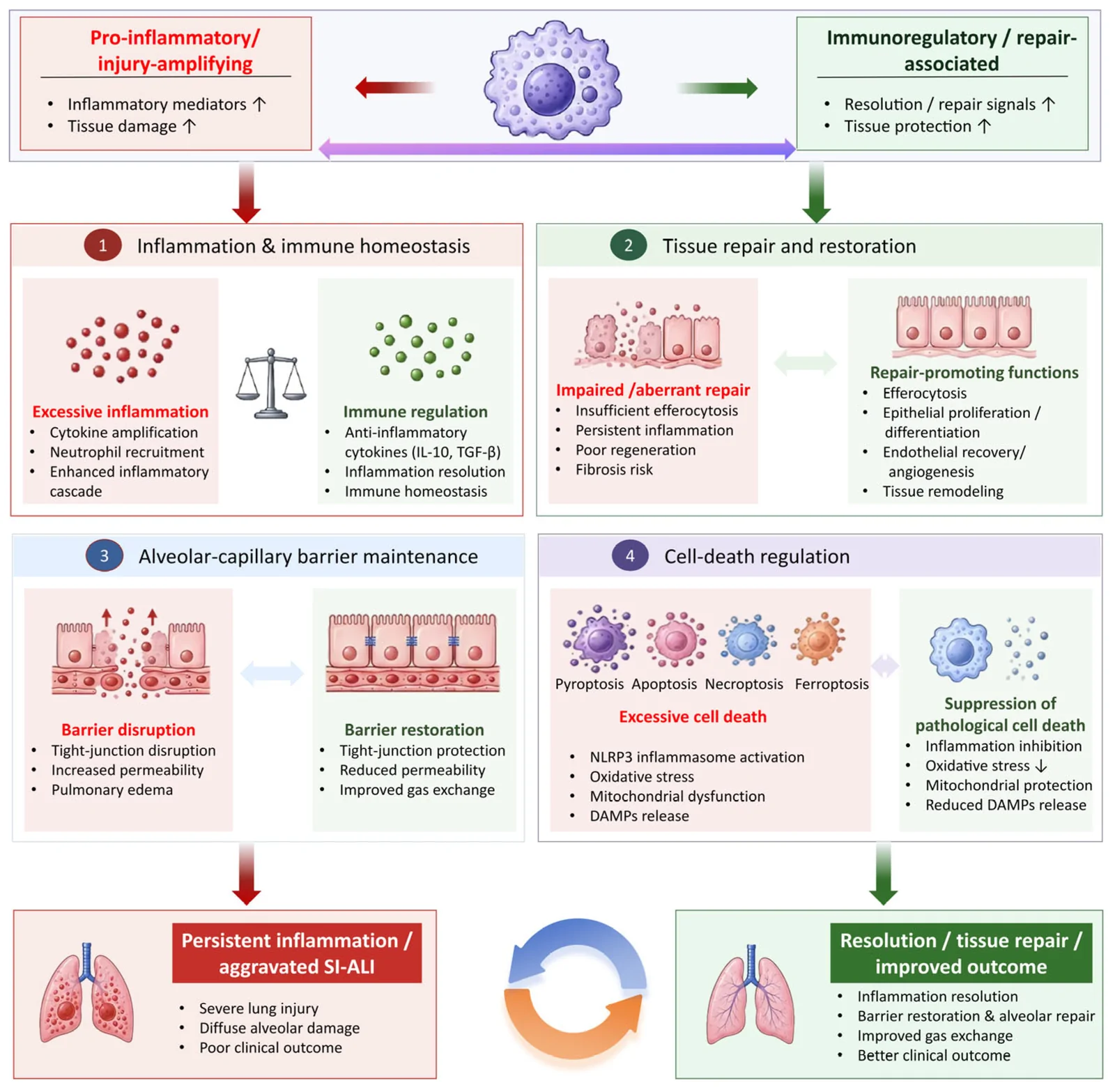
Macrophage Reprogramming by EVs in SI-ALI: Inflammation, Repair, Barrier Function, and Cell Death. In the figure, red marks pro-inflammatory and injury-promoting processes, while green marks immune regulation and tissue repair. The downward arrows show progression toward worsening injury or recovery. Double-headed arrows indicate shifts between injury and repair, and the circular arrows reflect changes in SI-ALI over time.

EVs control the functional condition of macrophages by transporting different bioactive factors, thus further affecting the pathological course of SI-ALI. The biological impact of EVs is not only the promotion or inhibition of inflammatory reactions, but also includes various factors, including tissue repair, preservation of alveolar–capillary barrier integrity, and programmed cell death. All these functions have an impact on the progression of the disease and clinical outcomes. Thus, the multidimensional biological effects of EV-mediated macrophage polarization, including the roles of inflammation and immune regulation, tissue repair, maintenance of the alveolar–capillary barrier, cell death regulation, and the EV-macrophage axis in the development of SI-ALI, and the role of EV-mediated remodeling of macrophage activity in the development of SI-ALI and the evaluation of ARDS, will be summarized in this section as shown in Figure 5.

### 5.1. EV-Mediated Macrophage Polarization Maintains Immune Homeostasis

Inflammatory responses are indispensable for host defense during sepsis-induced ALI. While an appropriate inflammatory response facilitates pathogen elimination, excessive or persistent inflammation is a major driver of pulmonary tissue damage and organ dysfunction. Increasing evidence indicates that EVs maintain immune homeostasis by dynamically regulating macrophage polarization, thereby serving as critical mediators linking innate immune activation to inflammation resolution [122].

In the initial phases of SI-ALI, the release of EVs with pro-inflammatory cargo is triggered by the presence of pathogen-associated molecular patterns (PAMPs) and damage-associated molecular patterns (DAMPs). These EVs stimulate TLR4/NF-κB, MAPK, and NLRP3-related inflammasome-related signaling pathways, which facilitate M1 macrophage polarization and increase the production of pro-inflammatory cytokines, including TNF-α, IL-1β, and IL-6, which facilitates rapid antimicrobial responses [27,123,124]. Nevertheless, persistent M1 polarization enhances inflammatory processes, increases alveolar epithelial and endothelial damage, and eventually impairs pulmonary immune homeostasis. The disease progression is accompanied by a gradual increase in signaling associated with immune regulation and tissue repair, which does not imply the absence of the inflammatory response. The processes of pro-inflammatory, immunosuppressive, and repair-related processes can co-exist during the course of SI-ALI and are mutually controlled by the source of EVs and the local microenvironment. EVs secreted by mesenchymal stem cells, endothelial cells, and other reparative cell populations are enriched with immunoregulatory cargos, such as miR-150-5p, miR-92a, and HSPB8. These EVs induce M2 macrophage polarization by inhibiting NF-κB signaling and activating PI3K/Akt and STAT3 pathways, leading to the production of anti-inflammatory mediators (IL-10 and TGF-β) and the resolution of inflammation and restoration of immune homeostasis [125,126,127]. This active switch between M1- and M2-dominated macrophage responses not only prevents excessive inflammation but also creates a conducive microenvironment to further tissue repair.

Importantly, EV-mediated immune regulation is highly spatiotemporally coordinated. The biological effects of EVs are determined by their cellular origin, cargo composition, and the activation status of recipient cells. Consequently, EVs function not simply as pro-inflammatory or anti-inflammatory mediators, but rather as dynamic regulators that fine-tune macrophage polarization to balance antimicrobial immunity with tissue protection [128]. Collectively, EV-mediated regulation of macrophage polarization represents a fundamental mechanism for maintaining immune homeostasis throughout the progression of sepsis-induced ALI. Targeting key cargos in EVs and their coordinated regulatory networks may enable stage-specific modulation of inflammatory responses and provide novel opportunities for precision immunotherapy in sepsis-induced ALI [128].

### 5.2. EV-Mediated Macrophage Polarization Promotes Lung Tissue Repair

Restoration of pulmonary architecture is a critical component of recovery from sepsis-induced ALI. Effective tissue repair depends on timely resolution of inflammation, clearance of damaged cells, and regeneration of the alveolar structure. Increasing evidence indicates that EVs not only suppress excessive inflammation but also facilitate tissue regeneration by reprogramming macrophage polarization, thereby linking immune resolution with tissue repair [129].

With the resolution of inflammation, macrophages gradually switch to the M2 phenotype. In this transition, EVs secreted by mesenchymal stem cells (MSCs), alveolar epithelial cells, and endothelial cells are enriched with reparative miRNAs, proteins, and lipids. These bioactive cargos stimulate signaling pathways such as PI3K/Akt and STAT3, which boost the anti-inflammatory and repair activities of M2 macrophages. This leads to the amplification of production of repair-related mediators like IL-10 and TGF-β which form a microenvironment that facilitates pulmonary regeneration [130,131,132]. In addition to macrophage modulation, EVs also directly control structural cells in the lung. They facilitate the growth, translocation and differentiation of alveolar epithelial cells, endothelial cells and fibroblasts, thus enhancing tissue regeneration [133]. Specifically, MSC-derived EVs reduce oxidative stress, prevent apoptosis of alveolar epithelial cells, and promote proliferation and differentiation of alveolar type II epithelial cells, which helps to restore alveolar integrity [134]. Some EVs may also facilitate angiogenesis and enhance local microcirculation, which supports tissue repair with metabolic support [135].

Moreover, the tissue repair process also demands the preservation of a proper balance. The lack of regeneration leads to chronic tissue damage, and the overabundance or prolonged reparative processes can stimulate fibroblast activation and extracellular matrix deposition, which predisposes to pulmonary fibrosis. EV-induced macrophage polarization is thus a dynamic regulatory process that integrates inflammatory resolution with tissue regeneration to achieve balanced pulmonary structure and function recovery [136].

The regenerative capacity of EVs has generated considerable interest for therapeutic applications, particularly those derived from MSCs, which have demonstrated encouraging effects in improving lung pathology, promoting alveolar regeneration, and restoring pulmonary function. Nevertheless, several issues—including optimal treatment timing, dosage, and long-term safety—remain unresolved [137].

### 5.3. EV-Mediated Macrophage Polarization Preserves Alveolar–Capillary Barrier Homeostasis

The alveolar–capillary barrier is essential for efficient gas exchange and maintenance of pulmonary homeostasis. During sepsis-induced ALI, uncontrolled inflammation causes extensive damage to alveolar epithelial cells and pulmonary microvascular endothelial cells, disrupts intercellular junctions, increases vascular permeability, and ultimately leads to pulmonary edema and hypoxemia [138]. Increasing evidence indicates that EV-mediated macrophage polarization not only regulates the magnitude of inflammatory responses but also coordinates communication among immune cells, alveolar epithelial cells, and endothelial cells, thereby preserving alveolar–capillary barrier integrity [139].

In the early stages of inflammation, the sustained activation of M1 macrophages promotes the release of TNF-α, IL-1β, IL-6, and reactive oxygen species. These substances disrupt the epithelial and endothelial barrier activity and cause inflammatory cell infiltration and tissue edema [140,141]. EV-induced M2 macrophage polarization facilitates the generation of anti-inflammatory mediators, the re-expression of cell adhesion molecules, and the decrease in vascular permeability as inflammation subsides. It also facilitates functional recovery of alveolar epithelial cells and endothelial cells, thus improving barrier integrity [142,143]. In addition to immunomodulatory actions, EVs directly control structural cells in the lung by enhancing cell survival, stabilizing intercellular junctions, and promoting cytoskeletal remodelling [144]. In particular, EVs derived from mesenchymal stem cells can alleviate inflammation-induced apoptosis and enhance alveolar fluid clearance. They also recover pulmonary vascular permeability to normal levels and enhance the recovery of lung barrier function [145].

The homeostasis of alveolar–capillary barrier depends on the concerted actions of immune cells, epithelial cells, endothelial cells, and the pulmonary microvasculature instead of on single signaling pathways. Through the coordination of signal transduction between these cellular compartments, EVs have the potential to inhibit inflammatory damage and stimulate structural repair, thus facilitating the progressive recovery of barrier integrity and lung function. It has been noted that EV-mediated immune regulation can decrease the occurrence of secondary pulmonary fibrosis and chronic respiratory dysfunction after ALI [146].

### 5.4. Regulation of Programmed Cell Death and Tissue Injury

Programmed cell death is a fundamental process governing tissue homeostasis and inflammatory responses, and its dysregulation contributes substantially to the pathogenesis of sepsis-induced ALI. Related studies have shown that EVs play an important role in the inflammatory amplification and tissue damage associated with SI-ALI by modulating the functional state of macrophages and influencing cell death-related signaling pathways [147].

In the inflammatory microenvironment, EVs derived from injury-associated cells can enhance the inflammatory activation of macrophages and participate in the regulation of inflammatory cell death. For example, some inflammation-associated EVs can deliver specific miRNAs, proteins, and DNA-related danger signals, thereby activating signaling pathways such as NF-κB and the NLRP3 inflammasome. This, in turn, promotes caspase-1 activation and the maturation and release of inflammatory mediators such as IL-1β and IL-18, thereby enhancing the inflammatory response of macrophages and contributing to pyroptosis-related inflammatory amplification. In addition, danger signals such as mtDNA carried by some EVs can be recognized by TLR9 and DNA-sensing pathways such as cGAS-STING. Consequently, they promote the expression of inflammation-related genes and the activation of the NLRP3 inflammasome, thereby contributing to inflammation amplification and tissue damage during the SI-ALI process.

Conversely, EVs with immunomodulatory effects can reduce the level of abnormal cell death by suppressing inflammatory signaling, alleviating oxidative stress, and maintaining mitochondrial function. For example, MSC-derived EVs can reduce inflammatory damage and improve the functional status of recipient cells by delivering anti-inflammatory miRNAs, proteins, and mitochondrial-related components.

In summary, EV-mediated macrophage polarization not only determines the severity of the inflammatory response but also synergistically regulates the signaling pathways involved in programmed cell death. At the same time, therapeutic strategies targeting both EV components and cell death can produce synergistic effects by suppressing inflammation, maintaining cell survival, and promoting tissue regeneration.

### 5.5. The Role of EV-Mediated Modulation of Macrophage Functional Remodeling in the Progression of SI-ALI and the Assessment of ARDS Risk

Persistent worsening of SI-ALI can progress to ARDS, the development of which is closely associated with the continued amplification of pulmonary inflammation and damage to the alveolar–capillary barrier [148,149]. During sepsis, persistent pathogen-associated signals and tissue damage signals can sustain the pulmonary inflammatory response and promote the activation of macrophages and other immune cells [150]. Subsequently, the release of large amounts of pro-inflammatory cytokines and chemokines further promotes the recruitment of neutrophils and monocytes, exacerbating damage to the alveolar epithelium and vascular endothelium. As the integrity of the alveolar–capillary barrier declines and vascular permeability increases, protein-rich fluid enters the alveoli, leading to pulmonary edema and a reduction in the effective gas exchange surface area. When the aforementioned inflammation and barrier damage continue to worsen, significant gas exchange impairment and hypoxemia may develop, reaching the clinical severity of ARDS.

This pathological progression overlaps significantly with the process described earlier, in which EVs mediate macrophage functional reprogramming to influence SI-ALI. Enhanced pro-inflammatory function of macrophages promotes the release of inflammatory mediators, neutrophil recruitment, and damage to alveolar epithelium and vascular endothelium, while EVs can modulate these macrophage responses through the functional cargo they carry. Previous studies have shown that EVs derived from patients with septic shock can be taken up by alveolar macrophages and promote pro-inflammatory activation of macrophages and lung injury through the EV-S100A8/A9–RAGE signaling pathway. Another study found that miR-223-3p in plasma EVs from patients with sepsis can act on alveolar macrophages to promote inflammatory responses, autophagy, and ferrocytosis, and is associated with septic ARDS. Therefore, EV-mediated macrophage functional remodeling may be involved in the sustained amplification of inflammation and the exacerbation of lung tissue damage in SI-ALI, thereby linking it to the pathophysiological process of ARDS.

EVs and their cargo are increasingly attracting attention as candidate biomarkers for reflecting disease severity and the progression of lung injury. Clinical studies have shown that plasma exosome levels in patients with sepsis increase with disease severity and are associated with SOFA scores, septic shock, and the risk of death. CD63 levels in exosomes are also associated with the degree of organ dysfunction and the risk of death, suggesting that exosomes can, to some extent, reflect the severity of sepsis [151]. Furthermore, the composition of EVs can change with the onset and progression of ARDS [152]. Clinical studies have found that elevated caspase-1 activity in circulating EVs in patients with sepsis is associated with the development of ARDS. Furthermore, the decline in EV miR-126-3p and miR-126-5p levels from Day 1 to Day 3 after admission is associated with the development of ARDS, suggesting that the dynamic changes in various cargoes within EVs may have value in assessing disease progression and prognosis. More direct evidence indicates that EV-S100A8/A9 levels are elevated in sepsis patients who develop ARDS and demonstrate a certain predictive capacity for ARDS. The same study further found that EVs derived from patients with septic shock can be taken up by alveolar macrophages and, through the S100A8/A9–RAGE axis, promote pro-inflammatory activation of macrophages and lung injury. Another study found that miR-122-5p, miR-223-3p, and LCN2 in plasma EVs were all identified as independent predictors of septic ARDS. Among these, EV-miR-223-3p can also act on alveolar macrophages, promoting inflammatory responses, autophagy, and ferrocytosis through MEF2C/Hippo-related signaling pathways.

In summary, EV-mediated macrophage functional reprogramming contributes to the amplification of inflammation and the progression of lung injury in SI-ALI, and the associated EVs and their cargoes may vary with disease severity and the onset of ARDS. Therefore, the characteristics of EVs associated with macrophage activation may serve as potential biomarkers for reflecting the progression of SI-ALI and assessing the risk of ARDS.

## 6. The Therapeutic Potential and Challenges of Extracellular Vesicles in Sepsis-Induced Acute Lung Injury

Currently, the treatment of SI-ALI primarily includes antimicrobial therapy, source control, organ support, and lung-protective therapy [153,154]. Among these, infection control forms the foundation of treatment. For clinically common bacterial sepsis, appropriate antibiotic therapy is an indispensable intervention [155]. If the infection is caused by other pathogens, antimicrobial therapy should be administered according to the specific pathogen. In addition, once the site of infection has been identified, prompt control of the source of infection is required, along with necessary organ support and lung-protective therapy based on the patient’s condition.

Apart from antimicrobial and supportive therapies, pharmacological treatments targeting abnormal inflammatory responses have also attracted attention. Glucocorticoids bind to glucocorticoid receptors, regulate the transcription of inflammation-related genes, and inhibit pro-inflammatory signaling pathways such as NF-κB and AP-1. Besides genomic effects [156], glucocorticoids also exert non-genomic effects that may influence mitochondrial function [157,158]. Clinical studies have evaluated its therapeutic efficacy in ARDS, and relevant guidelines recommend its use in certain patients with septic shock and ARDS [154,159]. In addition, targeted therapies for TNF-α and IL-6 have also been explored. Monoclonal antibodies such as adalimumab (anti-TNFα activity) and tocilizumab (anti-IL-6 activity) have both demonstrated anti-inflammatory effects in experimental ALI models and can reduce lung tissue damage [160]. Tocilizumab has also shown similar lung-protective effects in a CLP-induced sepsis model [161].

**Figure 6:**
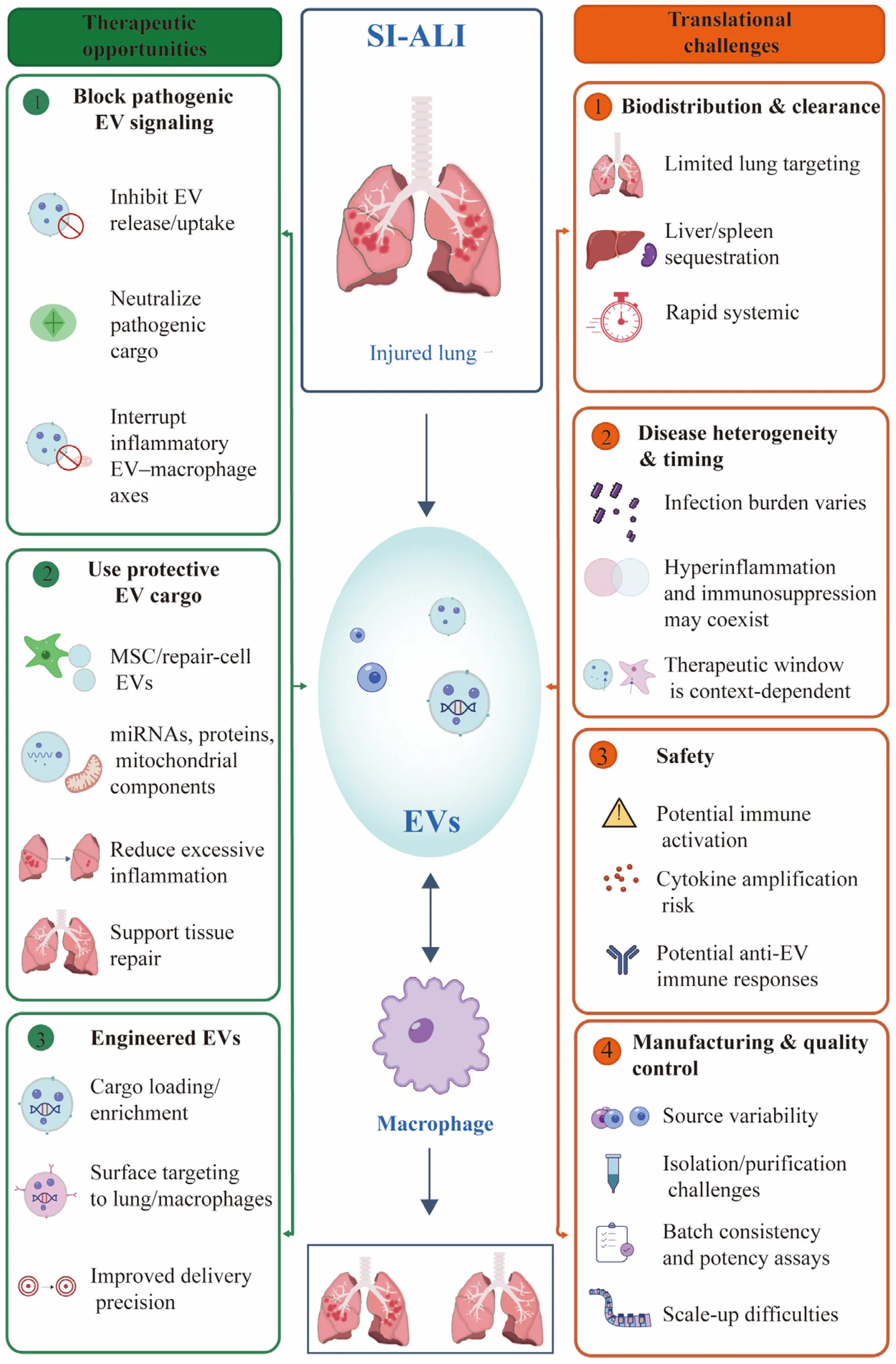
Therapeutic Opportunities and Translational Challenges for Cargo in EVs in SI-ALI. In the figure, the blue downward arrows indicate the direction of progression of the pathological process or treatment, while the double-headed arrows indicate bidirectional interactions between EVs and macrophages.

Thus, despite the availability of different treatment methods to address SI-ALI, there is a lack of specific interventions to address complex immune imbalances. With the growing understanding of the mechanisms that regulate EV-mediated macrophage functional reprogramming, therapeutic strategies that prevent pathogenic EV signaling and exploit protective EVs and engineered EVs to deliver therapeutic genes and proteins to specific locations are slowly becoming a focus. Nevertheless, therapeutic efficacy of various EV strategies depends on the cell origin, cargo, in vivo distribution, and disease status. Moreover, their engineering presents some difficulties in terms of safety and quality control. To overcome these challenges, the subsequent sections will cover the therapeutic potential of EVs and their functional cargo, engineering of EVs and targeted delivery, and the key challenges in clinical translation, as illustrated in Figure 6.

### 6.1. The Therapeutic Potential of EV and Functional Cargo

Currently, the treatment of SI-ALI remains centered on infection control, organ support, and lung-protective therapies [162]; interventions targeting EV-mediated immune regulation are still in the preclinical research stage. Therefore, EV-related therapies are better suited as potential supplements to the existing treatment regimen rather than as substitutes for anti-infective therapies. Regarding the interaction between EVs and macrophages, current intervention strategies primarily focus on two approaches. The first involves blocking pathogenic EV signaling, while the second involves utilizing protective EVs and their functional components. As the mechanisms by which EVs mediate macrophage functional remodeling become increasingly clear, therapeutic research has gradually shifted from simply altering macrophage polarization to blocking specific pathogenic EV signals and utilizing protective EV components. Currently, evidence supporting these therapeutic approaches is primarily focused on exosomes, particularly those derived from mesenchymal stem cells and their functional RNA, protein, and mitochondria-related components. In contrast, therapeutic research on microvesicles and apoptosomes remains relatively limited.

Exosomes derived from MSCs and other regenerative cells can carry molecules with protective immune effects, offering an alternative therapeutic approach for restoring macrophage function. Currently, the most compelling evidence pertains to RNA in exosomes. For example, miR-7704 in MSC-derived exosomes can influence pulmonary macrophage function by regulating the MyD88/STAT1 signaling pathway, thereby improving the clinical course of ALI. miR-125b-5p and miR-223 in exosomes derived from BMSCs, as well as mitochondrial-related components in EVs derived from ADMSCs, are also associated with inhibiting macrophage pyroptosis, alleviating inflammatory responses, and restoring mitochondrial function, respectively. These studies suggest that EV therapy can be further extended from the use of intact vesicles to the screening and enrichment of functional components within them that possess well-defined protective effects.

On the other hand, in the SI-ALI inflammatory environment, EVs from different sources can elicit different or even opposite immunological effects. For pathogenic EVs released by inflammation-associated cells, intervention can be achieved by inhibiting the formation, release, and intracellular action of key functional components. For example, miR-30d-5p from neutrophil-derived exosomes can enhance the inflammatory response and pyroptosis of macrophages via the SOCS1/SIRT1-NF-κB/NLRP3 pathway. However, administration of a miR-30d-5p inhibitor can reduce the activation and cell death of inflammatory macrophages in the lungs. Similarly, miR-92a-3p from exosomes derived from alveolar epithelial cells can also promote the inflammatory activation of alveolar macrophages; inhibiting its expression attenuates this pro-inflammatory effect. Therefore, compared with nonspecific inhibition of inflammation, targeting signaling pathways associated with specific EV components will be a more effective therapeutic strategy.

### 6.2. Engineered EVs and Targeted Delivery

The therapeutic efficacy of natural EVs may be limited by factors such as the concentration of the protective cargo, batch-to-batch variability, and nonspecific distribution within the body. The therapeutic molecules they carry are typically loaded through the cells’ own biogenesis processes; since the content of some active components is limited, higher doses may be required to achieve a stable therapeutic effect. At the same time, native EVs have limited tissue-targeting capabilities; following intravenous administration, they may accumulate in off-target tissues and be rapidly cleared. This reduces the effective dose reaching the target tissues and receptor cells [163]. Therefore, using engineering approaches to improve the loading efficiency of therapeutic components and enhance the selective delivery of EVs to lung tissue and macrophages has become a key strategy for improving the controllability of EV therapy.

Therapeutic cargo loading and EV surface modification are the main aspects of EV engineering nowadays. The latter can be done by modulating, transfecting, or transducing donor cells before EV formation, thus enriching certain molecules—including RNA and proteins—in the EV generation process. It may also be accomplished following EV isolation through loading exogenous therapeutic molecules through incubation, electroporation, or sonication. The latter method consists of the addition of proteins, peptides, glycan chains, or other targeted ligands to the EV surface to increase their binding and uptake efficiency by particular tissues or receptor cells. Thus, the choice of various engineering strategies must be based on a thorough evaluation of the properties of the therapeutic molecule, its target sites, and anticipated in vivo distribution properties [164].

For SI-ALI, the significance of engineered EVs lies not merely in increasing the number of EVs themselves, but in matching functional components with well-defined immunomodulatory effects to target macrophages. Therefore, priority should be given to enriching or exogenously loading miRNAs, proteins, or other regulatory molecules with established protective effects. This should be combined with targeted modifications for the lungs or macrophages to enhance their effective delivery within the local inflammatory microenvironment. The lipid bilayer of EVs can also, to a certain extent, protect the internal RNA, DNA, and proteins from enzymatic degradation in the circulatory environment, providing a carrier basis for the intracellular delivery of therapeutic molecules.

In this light, the advantage of engineered EVs is not simply a matter of enhancing the biological effects of natural EVs. This involves optimizing the composition and concentration of the functional cargo and enhancing its efficient delivery to specific receptor cells. However, validation of these strategies in SI-ALI is currently limited primarily to preclinical studies, and their actual therapeutic value remains dependent on various factors, including in vivo distribution, uptake by target cells, and the disease microenvironment.

### 6.3. Challenges in Clinical Translation

Both natural and engineered EVs have shown some therapeutic potential. However, their clinical translation is still limited by factors such as pharmacokinetics, disease heterogeneity, immunological safety, and product standardization. The specific analysis is as follows.

To begin with, EVs that are given systemically do not build up in the lungs. On the other hand, EVs injected intravenously may be deposited in various organs, such as the liver, lungs, kidneys, and spleen, and are eliminated in the systemic circulation in a few hours [165]. This indiscriminate distribution decreases the effective dose that reaches target macrophages and exposes non-target tissues. Thus, future research must not only assess whether EVs penetrate lung tissue but also provide additional explanations of whether they can penetrate certain groups of pulmonary macrophages.

Second, the dynamic and complicated immune condition of SI-ALI makes it difficult to choose an optimal treatment window. In sepsis, there is no strictly determined chronological order of inflammation, immunosuppression, and tissue repair processes [166,167]. Thus, one kind of EV with anti-inflammatory or pro-repair properties might not be applicable to all patients or all disease stages. In the case of an infection that has not been successfully suppressed yet, the over-inhibition of macrophage inflammation and phagocytic activity can lead to the inability to clear the pathogen. On the other hand, in cases where chronic pulmonary inflammation and tissue damage are the predominant features, the inhibition of pathogenic EV signaling or the provision of protective factors is a more promising treatment option. Thus, EV therapy should be timed according to the infection status and immune condition of the patient, and not just with the aim of promoting the M1-to-M2 shift as a standard therapeutic objective.

Furthermore, EVs are not entirely immune to the inert carrier. Their immunological effects are influenced by the source and internal components of the donor cells, and repeated administration may also trigger other immune responses. Existing studies indicate that repeated administration of engineered EVs leads to the production of EV-specific IgG, suggesting that EVs do not possess absolute immune privilege. For patients with sepsis who are in a state of severe inflammation, it is particularly important to evaluate whether EVs, under different dosages, routes of administration, and repeated dosing conditions, may further enhance innate immune activation or cytokine release.

Lastly, there are still difficulties with mass production of EV products. The quantity and the molecular composition of EVs can be changed by differences in donor cells, culture conditions, isolation, purification, and storage methods. Moreover, these factors can cause problems with loading efficiency, EV aggregation, membrane structure changes, purification losses, and decreased reproducibility [168,169]. Trade-offs between various engineering methods are also evident. As an illustration, endogenous loading is more favorable to the stable incorporation of nucleic acids and proteins. It is however constrained by the state of the donor cells and production efficiency [170]. Post-isolation loading is more flexible but can lead to the loss of EV membrane integrity and the necessity of further purification. Thus, in the case of EV therapeutic strategies based on particular functional components, the quality control in the future should not be reduced to the size of particles, concentration, and conventional surface markers, but the content, loading efficiency, and biological potency of the main therapeutic molecules should be clearly defined to guarantee the therapeutic efficacy of the product.

Overall, the application of EV therapy in SI-ALI is still in the transition phase from mechanistic research to precise delivery. Engineering modifications can, to some extent, enhance the loading and targeting capabilities of therapeutic components. However, challenges such as the pharmacokinetics of EVs, immunological safety, and standardization of production remain to be addressed. In the future, we will screen for EV components with defined biological effects based on the immune status of SI-ALI patients and combine them with targeted delivery to the lungs or macrophages. This approach aims to achieve precise treatment of abnormal inflammation and tissue damage without compromising pathogen clearance.

## 7. Conclusions

As key mediators of intercellular communication, EVs play important roles in immune regulation and the maintenance of tissue homeostasis in SI-ALI. This review systematically summarizes the molecular mechanisms through which distinct EV subtypes and their functional cargoes, including RNAs, proteins, lipids, and DNA, regulate macrophage functional states. By transferring diverse bioactive cargoes, EVs can induce multiple forms of macrophage functional reprogramming, including but not limited to M1/M2-associated phenotypic changes. These impacts are influenced by various factors, such as the cellular source of EVs, contents of EVs, and the microenvironment associated with diseases. Specific emphasis is placed on the signaling pathways related to NF-κB, PI3K/Akt, STAT3, SIRT1/SOCS1, and the NLRP3 inflammasome. In addition, we explore how EV-induced reprogramming of macrophages regulates inflammatory and immune balance, alveolar–capillary barrier integrity, tissue regeneration, and cell death. Overall, the above information provides theoretical evidence to develop immunoregulatory treatments based on EVs for SI-ALI.

Although there have been great improvements in immune modulation via EVs, there still exist certain questions that need further investigation. For instance, it is still unknown how the specific types of EVs and their cargoes influence the functional states of macrophages alone or through collaboration and how the regulatory processes develop dynamically throughout the course of SI-ALI development. It seems possible that the combination of single-cell sequencing, spatial transcriptomic methods, multiomics, and engineered EV technology could shed some light on this topic. In addition, EV-based therapeutic strategies continue to face major challenges related to targeted delivery efficiency, immunological safety, manufacturing standardization, and clinical translation.

## Data Availability

No new data were created or analyzed in this study. Data sharing is not applicable to this article.
